# 离子色谱-质谱联用技术在生命健康领域中的应用进展

**DOI:** 10.3724/SP.J.1123.2023.11001

**Published:** 2024-10-08

**Authors:** Yuxin YI, Micong JIN

**Affiliations:** 宁波市疾病预防控制中心, 浙江省微量有毒化学物健康风险评估技术研究重点实验室, 浙江 宁波 315010; Ningbo Municipal Center for Disease Control and Prevention, Key Laboratory of Health Risk Appraisal for Trace Toxic Chemicals of Zhejiang Province, Ningbo 315010, China

**Keywords:** 离子色谱-质谱联用, 食品分析, 药物分析, 代谢组学, 临床中毒分析, 综述, ion chromatography-mass spectrometry (IC-MS), food analysis, drug analysis, metabolomics, clinical poisoning analysis, review

## Abstract

离子色谱是一种用于分离强极性和可电离物质的色谱技术,质谱是一种基于离子*m/z*的分析技术,离子色谱与质谱联用(IC-MS)可用于复杂样品中可电离化合物(包括无机化合物和有机化合物)的分离、鉴定与定量。本文综述了IC-MS技术的发展以及近20年来IC-MS技术在食品分析、药物分析、代谢组学和临床中毒检测等领域的应用进展和未来发展趋势。在食品分析方面,本文主要介绍了IC-MS技术在无机阴离子、有机酸、极性农药、生物胺和糖类等物质检测方面的应用进展;在药物分析方面,本文主要介绍了IC-MS技术在药物杂质分析、药物降解产物鉴定和血药浓度测定领域的研究进展;在代谢组学方面,本文主要介绍了IC-MS技术在生物基质中的有机酸、糖磷酸酯、核苷酸等强极性代谢物分离、分析领域的研究进展;在临床中毒分析方面,本文主要介绍了IC-MS技术在烷基甲基膦酸、甲基膦酸、草甘膦、3-硝基丙酸和茚满二酮类鼠药等强极性或可电离有毒化合物检测方面的应用进展。本文将为IC-MS技术在生命健康领域的进一步拓展和应用提供有益参考。

## Abstract (en)

Ion chromatography is a technique commonly used to separate strongly polar and ionizable substances; it can be used to separate, identify, and quantify ionizable compounds in complex samples when coupled with mass spectrometry, and is currently being used in the application of food analysis, drug analysis, metabolomics and clinical poisoning analysis. Herein, we review the development of ion chromatography-mass spectrometry (IC-MS), its progress over the past 20 years, and future trends in the abovementioned areas. The IC-MS research progress and applications for the determination of inorganic anions, organic acids, polar pesticides, biogenic amines, and sugars in the food field are discussed. Drug analysis applications are discussed mainly in relation to the analysis of drug impurities, identifying drug degradation products, and determination of plasma concentration, while the separation and analysis of strongly polar metabolites, such as organic acids, sugar phosphates, and nucleotides in biological matrices are discussed in relation to metabolomics. Advances in the analysis of strongly polar or ionizable toxic compounds, such as alkyl methylphosphonic acid, methylphosphonic acid, glyphosate, 3-nitropropionic acid, and indandione rodenticides, are mainly discussed in clinical poisoning analysis field. This paper is expected to become a useful reference for the further expansion and application of IC-MS in the life and health fields.

离子色谱是一种基于离子交换原理的分离技术,可选择性地分离阳离子或阴离子,通过调节流动相的pH、离子强度等参数,离子色谱可实现对不同离子化合物的选择性分离;质谱是一种基于离子*m/z*的分析技术,其将离子化合物转化为气相离子,并通过质谱仪中的离子源、质量分析器和检测器等部件来进行离子化和质谱分析,从而实现对未知化合物的定性和定量分析。离子色谱-质谱联用(IC-MS)技术是离子色谱和质谱两种分析技术的有效结合,通过离子色谱可对样品中的强极性和可电离物质进行分离,再通过质谱仪进行定性和定量分析,从而实现复杂基质样品中强极性和可电离物质的高效分离和准确检测。针对强极性和可电离物质,IC-MS技术无需衍生化即可实现分离和检测,具有高灵敏度、高选择性的优点,能够将基质干扰降至最低^[1]^。IC-MS技术的早期应用主要集中在环境监测领域,目前已有越来越多的IC-MS方法被开发出来,并应用于生命健康领域,其中包括医药分析和食品安全等^[2]^。本文主要对IC-MS技术在生命健康领域(食品分析、药物分析、代谢组学和临床中毒分析等方面)中的应用进展进行综述,以期为相关领域的研究和应用提供有益参考。

## 1 IC-MS技术

IC-MS技术主要包括离子色谱-高分辨质谱(IC-HRMS)^[3]^、毛细管离子色谱-串联质谱(Cap IC-MS/MS)^[4]^、离子色谱-一级质谱(IC-MS^1^)和离子色谱-串联质谱(IC-MS/MS)^[5]^以及离子色谱-电感耦合等离子体质谱(IC-ICP-MS)^[6]^等。Cap IC-MS/MS技术常用于小分子、蛋白质和肽的分析;IC-ICP-MS常用于多组分、可电离化合物的定性和定量分析,其中ICP电离源的电离效率通常高于电喷雾电离(ESI)源,因此IC-ICP-MS也是元素形态分析的首选技术,常用于砷、硒、汞、铬等的形态分析^[7]^。

离子色谱的淋洗液中往往含有非挥发性盐,尽管通过串接抑制器可去除大部分无机盐,但将离子色谱与质谱联用时,仍会有部分无机盐进入质谱离子源,导致离子源中产生无机盐沉积和喷雾针堵塞等问题。因此,接口问题是IC-MS技术中需要解决的一个关键问题。若仅从无机盐的角度考虑,色谱柱内径越小且流动相流速越慢就越有利于减少质谱仪中淋洗液的进入量,从而减少无机盐在离子源中的沉积;但当色谱柱内径变小后,进样量也会减少,从而可能导致微量组分无法检出。对于相同进样体积的样品,与常规分析柱相比,使用毛细管色谱柱能够获得更高的灵敏度和更高的分离效率,同时毛细管色谱柱还具有耗样低的优势。1983年,Rokushika等^[8]^使用内径较小的毛细管离子色谱柱结合阳离子型中空纤维膜抑制器,实现了多种低含量无机阴离子和有机酸的分离和检测,这也是首次报道的一种抑制型毛细管离子色谱技术。近年来,离子色谱中的常规分析柱逐渐向窄径柱和毛细管柱方向发展,以此来满足复杂样品的多组分分离和质谱接口的需求。为了提高质谱检测的灵敏度和喷雾稳定性,可通过调整离子源中的喷雾器气体流量和温度等参数来增强去溶剂化效应,也可以通过添加挥发性溶剂来优化淋洗液的性质,以使柱后流出液能够与质谱更好地兼容。与此同时,质谱也经历了一级质谱、串联质谱和高分辨质谱等不同发展阶段,其中在分析鉴定方面,高分辨质谱可最大限度地减少来自色谱共流出物的干扰,具有更高的分析选择性和灵敏度^[9]^。

## 2 IC-MS技术的应用

### 2.1 食品分析

#### 2.1.1 无机阴离子

IC-MS技术常应用于食品中溴酸根、高氯酸根和氯酸根等无机阴离子的检测。溴酸根的检测可以采用IC-ICP-MS和IC-MS/MS两种方式。IC-ICP-MS具有很高的灵敏度和选择性,可以特异性地测定溴酸根,如Heitkemper等^[10]^利用IC-ICP-MS技术测定面包中的溴酸盐,所得到的检出限(LOD)为6 μg/kg; Akiyama等^[11]^同样采用此种方法对溴酸盐进行测定,所得到的LOD为2 μg/kg。韩梅等^[12]^以30 mmol/L NH_4_NO_3_(pH 8.0)作为淋洗液,利用IonPac AS19色谱柱(250 mm×4 mm)进行分离,建立了一种同时检测饮用水中溴酸根和溴离子的IC-ICP-MS分析方法,在最佳实验条件下溴酸根和溴离子的LOD分别为0.25 μg/L和0.20 μg/L。此外,Aggrawal等^[13]^开发了一种基于IC-MS^1^测定面粉及其制品中溴酸盐含量的分析方法,采用高性能的IonPac AS31阴离子交换柱(250 mm×2 mm)进行分离,大体积进样,所得到的溴酸盐定量限(LOQ)为17 μg/kg。为了进一步提高检测灵敏度,张萍等^[14]^采用IC-MS/MS方法,以IonPac AS20色谱柱(250 mm×2 mm)为分析柱、KOH溶液为淋洗液,在多反应监测(MRM)模式下实现了牛奶中高氯酸盐、溴酸盐和碘离子的快速测定,其中溴酸盐的LOD为0.1 μg/L。

离子色谱电导检测器的灵敏度和选择性存在一定局限性,同时,作为持久性污染物,氯酸盐和高氯酸盐在食品中的残留量一般较低,对二者进行测定时需搭配具有更高灵敏度和选择性的质谱检测器。Seyfferth等^[15]^以IonPac AS16色谱柱(250 mm×2 mm)为分析柱,利用NaOH溶液进行等度洗脱,开发了一种基于IC-MS^1^的植物组织中高氯酸盐分析方法,所得到的LOD为0.04 μg/L。在使用一级质谱测定高氯酸盐时,会出现一些*m/z*相近的离子(如H^34^S $O_{4}^{-}$ 、H^34^S^18^O^16^ $O_{3}^{-}$ 、H_2_P^18^O^16^ $O_{3}^{-}$ ),因此为了降低离子干扰及提高检测准确度,科学家们开发出了多种同位素内标串联质谱检测技术^[16⇓⇓⇓⇓-21]^。如Zhang等^[16]^研究了氯酸盐和高氯酸盐经3支离子色谱柱(IonPac AS11、IonPac AS16和IonPac AS19)分离后的色谱峰对称性,并基于IC-MS/MS建立了以IonPac AS16色谱柱(250 mm×2 mm)为分析柱的咖啡中氯酸盐和高氯酸盐快速测定方法。实验结果表明,氯酸盐的LOQ为15 μg/L,高氯酸盐的LOD为1.5 μg/L。El Aribi等^[17]^使用^18^O标记的高氯酸盐作为内标物,建立了食品和饮料中高氯酸盐的IC-MS/MS检测方法,该研究通过监测^35^Cl/^37^Cl的比值,提高了复杂样品中高氯酸盐测定的特异性和数据可靠性。朱伟等^[18]^以^18^O_3_标记的氯酸盐和^18^O_4_标记的高氯酸盐作为同位素内标,利用IonPac AS20色谱柱(250 mm×2 mm)进行分离,基于IC-MS/MS技术实现了婴幼儿配方奶粉中氯酸盐和高氯酸盐的含量测定,二者的LOD分别为1.0 μg/kg和0.3 μg/kg。上述方法均采用柱后抑制器除盐的操作,但不论如何除盐,总会有少量的无机盐进入质谱离子源,从而导致进样毛细管的污染或堵塞。为此,封丽娟等^[19]^将甲胺水溶液和乙腈作为流动相,采用梯度洗脱的方式,建立了无需除盐的IC-MS/MS方法,并实现了面制品中溴酸盐、氯酸盐和高氯酸盐的测定,三者的LOD分别为0.002、0.02和0.002 mg/kg。与传统的柱后抑制器除盐操作相比,该方法极大地减少了阴、阳离子对离子源的污染以及淋洗液所产生的背景噪声,同时避免了非挥发性碱对质谱检测器造成的伤害。IC-MS技术在无机阴离子检测领域的应用总结见表1。在现有报道的无机阴离子检测方法中,以KOH溶液作为淋洗液的IC-MS/MS检测技术较为成熟,重复性较好,这也是目前无机阴离子定量检测的主要方法。然而,淋洗液中无机盐的存在仍是导致质谱离子源污染或毛细管堵塞的主要原因,因此开发探索可挥发淋洗液将是IC-MS技术未来的一个重要研究方向。

**表1:** IC-MS技术在无机阴离子检测中的应用

Analytes	Sample types	IC separation column	Elution solvents and elution program	LODs	LOQs	Method
Bromate	bread	IonPac AS10 (250 mm×4 mm)	NaOH solution; gradient elution	6 μg/kg	-	IC-ICP- MS^[10]^
Bromate	bread	GL-IC-A15	(NH_4_)_2_CO_3_ solution; isocratic elution	2 μg/kg	5 μg/kg	IC-ICP- MS^[11]^
Bromate and bromide	drinking water	IonPac AS19 (250 mm×4 mm)	NH_4_NO_3_ solution; isocratic elution	bromate: 0.25 μg/L; bromide: 0.20 μg/L	-	IC-ICP- MS^[12]^
Bromate	flour products	IonPac AS31 (250 mm×2 mm)	KOH solution; gradient elution	5 μg/kg	17 μg/kg	IC- MS^1[13]^
Perchlorate, bromate and iodide	milk	IonPac AS20 (250 mm×2 mm)	KOH solution; isocratic elution	perchlorate: 0.02 μg/L; bromate: 0.1 μg/L; iodide: 0.5 μg/L	-	IC-MS/ MS^[14]^
Perchlorate	lettuce and spinach	IonPac AS16 (250 mm×2 mm)	NaOH solution; isocratic elution	0.04 μg/L	-	IC- MS^1[15]^
Chlorate and perchlorate	freshly brewed coffee	IonPac AS16 (250 mm×2 mm)	methylamine aqueous solution and acetoni- trile; gradient elution	-	chlorate: 15 μg/L; perchlorate: 1.5 μg/L	IC-MS/ MS^[16]^
Perchlorate	foods and beverages	IonPac AS20 (250 mm×2 mm)	KOH solution; gradient elution	0.5 ng/L	-	IC-MS/ MS^[17]^
Chlorate and perchlorate	infant formula milk powder	IonPac AS20 (250 mm×2 mm)	KOH solution; gradient elution	chlorate: 1.0 μg/kg; perchlorate: 0.3 μg/kg	chlorate: 3 μg/kg; perchlorate: 1 μg/kg	IC-MS/ MS^[18]^
Bromate, chlorate and perchlorate	flour products	IonPac AS16 (250 mm×2 mm)	methylamine aqueous solution and acetoni- trile; gradient elution	bromate: 0.002 mg/kg; chlorate: 0.02 mg/kg; perchlorate: 0.002 mg/kg	bromate: 0.007 mg/kg; chlorate: 0.07 mg/kg; perchlorate: 0.007 mg/kg	IC-MS/ MS^[19]^
Perchlorate	tea	IonPac AS20 (250 mm×2 mm)	KOH solution; isocratic elution	1.7 μg/kg	6 μg/kg	IC-MS/ MS^[20]^
Perchlorate	tea	IonPac AS19 (250 mm×2 mm)	KOH solution; gradient elution	0.3 μg/kg	1 μg/kg	IC-MS/ MS^[21]^

-: not mentioned. IC: ion chromatography; ICP: inductively coupled plasma.

#### 2.1.2 有机酸

有机酸是指具有一个或多个羧基官能团的有机化合物,食品中常见的有机酸包括乙酸、柠檬酸和苹果酸等,目前针对有机酸检测的主要方法是IC-MS^1^法^[22⇓⇓-25]^。周海波等^[22]^以IonPac AS20色谱柱(50 mm×2 mm)为分析柱,利用KOH溶液进行梯度洗脱,并基于IC-MS^1^建立了葡萄酒中柠檬酸的分析方法。Jin等^[23]^采用免试剂IC-MS^1^法,将IonPac AS11-HC色谱柱(250 mm×2 mm)作为分析柱,在选择离子监测(SIM)模式下同时测定了果汁和饮料中的20种有机酸,LOD为0.05~0.5 mg/L。王芳焕等^[24]^选用IonPac AS11-HC色谱柱(250 mm×2 mm)进行分离,利用KOH溶液进行梯度洗脱,基于IC-MS^1^建立了鲜榨果汁中6种*α*-羟基酸的测定方法,LOD为0.002~0.025 mg/L。Xiong等^[25]^建立了一种在线富集IC-MS^1^同时测定食品中16种有机酸的方法,该方法利用自制的富集柱和分离柱对有机酸进行在线富集和分离,使用NaOH溶液进行梯度洗脱,以负离子大气压化学电离源作为离子源,在SIM模式下实现了16种有机酸的定性和定量分析,所得LOD为0.01~0.22 mg/L。由于一级质谱的检测灵敏度较低且易受干扰,为进一步提高分析灵敏度和选择性,穆瑛琦等^[26]^基于IC-MS/MS技术建立了一种白酒、黄酒、干红葡萄酒中10种有机酸含量的测定方法,该方法无需衍生化处理,在ESI^-^源、MRM模式下测得10种有机酸的LOD为1.0~8.0 μg/L。IC-MS技术在有机酸检测领域的应用总结见表2。综合现有文献来看,食品中有机酸的测定主要以IC-MS^1^方法为主,但随着研究的不断深入和定性、定量检测要求的不断提高,IC-MS/MS技术将成为食品中有机酸测定的重要手段。

**表2:** IC-MS技术在有机酸检测中的应用

Analytes	Sample types	IC separation column	Elution solvents and elution program	LODs	LOQs	Method
Citric acid	wine	IonPac AS20 (50 mm× 2 mm)	KOH solution; gradient elution	17.6 μg/L	-	IC- M
Quinic acid, lactic acid, acetic acid, formic acid, iso-butyric acid, pyruvic acid, iso-valeric acid, valeric acid, malic acid, succinic acid, malonic acid, tartaric acid, maleic acid, oxalic acid, α-ketoglutaric acid, fumaric acid, citric acid, iso-citric acid, cis-aconitic acid and trans-aconitic acid	juices and beverages	IonPac AS11-HC (250 mm× 2 mm)	KOH solution (containing 10% methanol); gradient elution	0.05-0.5 mg/L	-	IC- MS^1[23]^
Malic acid, tartaric acid, citric acid, glycolic acid, lactic acid and mandelic acid	fresh juice	IonPac AS11-HC (250 mm× 2 mm)	KOH solution; gradient elution	malic acid, tartaric acid and citric acid: 0.002 mg/L; glycolic acid: 0.015 mg/L; lactic acid: 0.005 mg/L; mandelic acid: 0.025 mg/L	-	IC- MS^1[24]^
D-(-)-Quinic acid, L-pyroglutamate acid, lactic acid, formic acid, pyruvic acid, succinate acid, malic acid, propanedioic acid, D-tartaric acid, maleic acid, α-ketoglutaric acid, oxalic acid, fumaric acid, orthophosphoric acid, citric acid and trans-aconitic acid	fermented yoghurt, cereal beverage, chinkiang vinegar, fruit juice and red wine	Homemade separation column (250 mm× 1.0 mm)	NaOH solution; gradient elution	0.01-0.22 mg/L	0.03- 0.8 mg/L	IC- M
Oxalic acid, fumaric acid, maleic acid, malic acid, tartaric acid, citric acid, quinic acid, aconitic acid, succinic acid and lactic acid	liquor, yellow rice wine and dry red wine	IonPac AS11-HC (250 mm× 4 mm)	KOH solution; gradient elution	1.0-8.0 μg/L	3.5- 26.5 μg/L	IC-MS/ MS^[26]^

#### 2.1.3 极性农药

极性农药主要包括草甘膦及其代谢物(草铵膦、氨基甲基膦酸等)、乙烯利、2,4-二氯苯氧乙酸、氯酚等。Melton等^[27]^将IonPac AS19色谱柱(250 mm×2 mm)作为分析柱,基于IC-MS/MS技术同时测定了水果和蔬菜中的乙烯利、草铵膦和草甘膦等9种强极性农药,LOD为0.01~0.05 mg/kg。Bauer等^[28]^利用Metrosep A Supp 5色谱柱(150 mm×2 mm)柱进行分离、NH_4_HCO_3_溶液和乙腈进行梯度洗脱、同位素标记内标法进行校准,开发了一种不同植物性食品中乙烯利、氯酸盐、高氯酸盐和2-羟乙基膦酸等强极性农药的IC-MS/MS测定方法。Wang等^[29]^以IonPac AS20色谱柱(250 mm×4 mm)为分析柱、NaOH溶液为淋洗液,建立了一种同时测定茶叶中草甘膦、草铵膦和苯达松的IC-MS/MS方法,所得到的LOQ为0.36~1.30 μg/L。Bauer等^[30]^利用Metrosep A Supp 5色谱柱(150 mm×2 mm)进行分离、NH_4_HCO_3_溶液和乙腈进行梯度洗脱,基于同位素标记手段建立了植物基质中乙膦酸和膦酸的IC-MS/MS快速测定方法,所得到的LOD为10 μg/kg。Adams等^[31]^以酸化甲醇作为提取溶剂,利用IonPac AS19色谱柱(250 mm×2 mm)进行分离,基于IC-MS/MS技术建立了食品中草甘膦等11种农药及代谢物的同时测定方法,LOQ为0.01~100 mg/kg。卢思佳等^[32]^利用IonPac AS19色谱柱(250 mm×2 mm)进行分离,采用KOH溶液进行等度洗脱,在ESI^-^、MRM模式下同时测定了蔬菜中乙烯利和2,4-二氯苯氧乙酸,二者的LOD分别为0.2 μg/kg和0.05 μg/kg。为了进一步提高目标物的定性准确性,Chiesa等^[33]^开发了一种通用IC-HRMS方法,用于蜂蜜、鲈鱼和牛肌肉3种复杂基质中草甘膦、草铵膦和氨基甲基膦酸的检测,结果表明3种农药的LOQ为4.30~9.26 ng/g。Pareja等^[34]^利用甲醇水溶液或纯水对蜂蜜样品进行稀释,此过程中无需调节pH,随后利用IC-HRMS对蜂蜜样品中的草甘膦和氨基甲基膦酸进行测定,所得到的回收率为80%~110%。Nardin等^[35]^以甲酸和乙腈作为淋洗液,基于IC-HRMS技术建立了矿泉水、牛肉、果汁、茶叶等食品中阳离子季铵盐除草剂(百草枯、野燕枯、敌草快、矮壮素和助壮素)同时测定的方法,以评估这些食品的安全性。在氯酚检测方面,Jin等^[36]^建立了蛤蜊组织中14种痕量氯酚的IC-MS^1^测定方法,该方法使用80%甲醇水溶液(含5%三乙胺)作为提取溶剂,用超声辅助法进行快速提取,用Oasis HLB小柱进行固相萃取净化,采用IonPac AS11色谱柱(250 mm×4 mm)进行分离,并在SIM模式下实现了五氯酚等14种氯酚的定量测定。IC-MS技术在极性农药检测领域的应用总结见表3。

**表3:** IC-MS技术在极性农药检测中的应用

Analytes	Sample types	IC separation column	Elution solvents and elution program		LODs	LOQs	Method
Chlorate, ethephon, fosetyl-aluminium, glufosinate, glyphosate, N-acetyl aminomethyl phosphonic acid, N-acetyl glyphosate, perchlorate and phosphonic acid	fruit and vegetables	IonPac AS19 (250 mm× 2 mm)	KOH solution; gradient elution	-		-	IC-MS/ MS^[27]^
Chlorate, perchlorate, ethephon and 2-hydroxyethylphosphonic acid	plant-derived commodities	Metrosep A Supp 5 (150 mm×2 mm)	NH_4_HCO_3_ solu- tion and acetoni- trile; gradient elution	-		-	IC-MS/ MS^[28]^
Glyphosate, glufosinate and bentazone	tea	IonPac AS20 (250 mm× 4 mm)	NaOH solution; gradient elution	0.11-0.39 μg/L		0.36-1.30 μg/L	IC-MS/ MS^[29]^
Fosetyl and phosphonic acid	tomato, apple, lemon, sultana, avocado and wheat	Metrosep A Supp 5 (150 mm×2 mm)	NH_4_HCO_3_ solu- tion and acetoni- trile; gradient elution	-		10 μg/kg	IC-MS/ MS^[30]^
Glyphosate, aminomethyl phosphonic acid, N-acetyl aminomethyl phosphonic acid, glufosinate, 3-methylphosphinicopropionic acid, N-acetyl glufosinate, ethephon, chlorate, perchlorate, fosetyl aluminium and phosphonic acid	cereals, grapes and infant food	IonPac AS19 (250 mm× 2 mm)	KOH solution; gradient elution	-		0.01-100 mg/kg	IC-MS/ MS^[31]^
Ethephon and 2,4-dichlorophenoxyacetic acid	eggplant, tomato, corn and chili	IonPac AS19 (250 mm× 4 mm)	KOH solution; isocratic elution	ethephon: 0.2 μg/kg; 2,4- dichloropheno- xyacetic acid: 0.05 μg/kg		ethephon: 0.7 μg/kg; 2,4- dichloropheno- xyacetic acid: 0.17 μg/kg	IC-MS/MS^[32]^
Glyphosate, glufosinate and aminomethylphosphonic acid	honey, fish and bovine muscle	IonPac AS19 (250 mm× 2 mm)	KOH solution; gradient elution	-		4.30-9.26 μg/kg	IC-HRMS^[33]^
Glyphosate and aminomethylphosphonic acid	honey	IonPac AS19 (250 mm× 2 mm)	KOH solution; gradient elution	-		glyphosate: 0.005 mg/kg; aminom- ethyl phosphonic acid: 0.02 mg/kg	IC-HRMS^[34]^
Paraquat, difenzoquat, diquat, mepiquat and chlormequat	mineral water, beef, juice and tea	IonPac CS17 (15 mm× 2 mm)	formic acid, acetonitrile and water; gradient elution	chlormequat, difenzoquat and mepiquat: 1.5 μg/L; diquat and paraquat: 3 μg/L		chlormequat, difenzoquat and mepiquat: 5 μg/L; diquat and paraquat: 10 μg/L	IC-HRMS^[35]^
Pentachlorophenol, 2-chlorophenol, 4-chlorophenol, 3-chlorophenol, 2,6-dichlorophenol, 2,3-dichlorophenol, 2,5-dichlorophenol, 2,4-dichlorophenol, 3,4-dichlorophenol, 3,5-dichlorophenol, 2,3,5-trichlorophenol, 2,4,5-trichlorophenol, 2,4,6-trichlorophenol and 3,4,5-trichlorophenol	clam tissues	IonPac AS11 (250 mm× 4 mm)	KOH solution and acetonitrile; gradient elution	-		0.05-0.50 μg/kg	IC-MS^1[36]^

#### 2.1.4 生物胺

生物胺是一类含氮小分子有机化合物的统称^[37]^。常见的生物胺包括组胺、精胺以及色胺等,这些化合物在生物体内发挥着重要的生理功能。然而,食用含有高浓度生物胺的食物会对人体造成一些不良影响,严重时还会导致出现脑出血和死亡,因此生物胺的检测对人体健康具有重要的意义。Saccani等^[38]^将甲磺酸作为提取溶剂,采用IonPac CS17色谱柱(250 mm×2 mm)进行分离,建立了一种基于阳离子交换色谱的IC-MS^1^方法,并成功用于肉制品中尸胺、腐胺、组胺、胍丁胺、苯乙胺和亚精胺的同时测定。Š $\dot{c}$ avni $\dot{c}$ ar等^[39]^用纯水进行提取、IonPac CG17色谱柱(50 mm×4 mm)进行分离,建立了奶酪中10种生物胺的IC-MS/MS测定方法。Ko $\dot{c}$ ar等^[40]^基于IC-MS/MS技术建立了一种鲜鱼和加工鱼制品中10种生物胺的测定方法,该方法采用纯水对肌肉组织进行提取;与此同时,Ko $\dot{c}$ ar等^[41]^还分别使用IC-MS/MS、高效液相色谱以及半定量免疫测定试剂盒对鱼类产品中的生物胺进行检测,比较分析结果显示,所采用的3种方法具有良好的相关性。Zhu等^[42]^用IonPac CG17色谱柱(50 mm×4 mm)进行分离,基于IC-MS/MS技术,建立了一种葡萄酒中9种生物胺的简单、快速测定方法。此外,Suo等^[43]^使用75%乙腈甲醇溶液进行提取、C_18_固相萃取柱进行净化、IonPac SCS-1色谱柱(250 mm×4 mm)进行分离、同位素内标进行定量,并利用IC-MS/MS技术成功测定了血液和尿液中的胆碱、肉毒碱、乙酰肉毒碱(ACa)和乙酰胆碱(ACh)。IC-MS技术在生物胺检测领域的应用总结见表4。

**表4:** IC-MS技术在生物胺检测中的应用

Analytes	Sample types	IC separation column	Elution solvents and elution program	LODs	LOQs	Method
Cadaverine, putrescine, histamine, agmatine, phenethylamine and spermidine	fresh and processed meat	IonPac CS17 (250 mm× 2 mm)	methanesulfonic acid; gradient elution	9-34 μg/L	27-100 μg/L	IC-MS^1[38]^
Trimethylamine, putrescine, cadaverine, histamine, 2-phenylethylamine, spermine, spermidine, tryptamine, agmatine and tyramine	cheeses	IonPac CG17 (50 mm× 4 mm)	formic acid; gradient elution	12 μg/L- 5.4 mg/L	40 μg/L- 18 mg/L	IC-MS/MS^[39]^
Histamine, agmatine, cadaverine, putrescine, spermidine, spermine, tyramine, tryptamine, trimethylamine and 2-phenylethylamine	fresh fish and processed fish products	IonPac CG17 (50 mm× 4 mm)	formic acid; gradient elution	0.02-15.6 ng/g	-	IC-MS/MS^[40]^
Octopamine, putrescine, cadaverine, spermine, spermidine, tyramine, phen- ylethylamine, histamine and tryptamine	wine	IonPac CG17 (50 mm× 4 mm)	formic acid; gradient elution	0.6-500 μg/L	2-1700 μg/L	IC-MS/MS^[42]^
Choline, carnitine, acetylcarnitine (ACa) and acetylcholine (ACh)	blood and urine	IonPac SCS-1 (250 mm× 4 mm)	formic acid and acetonitrile; isocratic elution	-	choline and ACh: 0.2 mg/kg; carni- tine and ACa: 0.5 mg/kg	IC-MS/MS^[43]^

#### 2.1.5 糖类

糖类是食品中的常见成分,也是食品质量的重要指标,其中低聚糖是由3~20个单糖单元通过糖苷键连接而成的碳水化合物,而糖醇是醛糖或酮糖的羰基被还原成羟基后的衍生物。尽管糖醇不属于糖类化合物,但其具有糖的某些属性,因此糖醇常被用作食品工业中的甜味剂。周洪斌等^[44]^用纯水对米酒、醋、水果等多种食品样品中的糖醇进行提取,采用C_18_和混合离子交换固相萃取柱去除样品中的色素、有机酸和氨基酸等杂质,建立了食品中糖醇的IC-MS^1^检测方法,并在SIM模式下进行检测,所得到的LOQ为0.98~13.56 mg/kg。吴春敏等^[45]^采用CarboPac PA1色谱柱(250 mm×2 mm)进行分离,在ESI^-^源、MRM模式下进行监测,建立了同时快速测定婴幼儿配方乳粉及乳制品中半乳糖、葡萄糖、蔗糖和乳糖的IC-MS/MS方法。张涛等^[46]^将咖啡豆粉碎后加入去离子水进行超声提取,基于IC-MS/MS建立了咖啡豆样品中阿拉伯糖、半乳糖、葡萄糖、蔗糖、木糖、甘露糖、果糖和核糖的测定方法,该方法解决了不同碳含量糖类化合物之间的假阳性检测问题,适用于咖啡豆中典型单糖和小分子寡糖的同时测定。Fa等^[47]^通过对质谱条件进行优化,建立了一种Cap IC-MS/MS测定蓝藻细胞提取物中葡萄糖基甘油、蔗糖和其他碳水化合物的方法。但由于糖的种类较多,仅凭借串联质谱难以解决定性问题,因此Tian等^[48]^基于IC-HRMS技术,在葡萄渣中鉴定出了32种具有明确化学组成和分子结构的寡糖,并对其他61种未知物进行了分子结构的推测,初步认定这61种未知物均为寡糖;将该研究结果与使用液相色谱-质谱(LC-MS)所获得的数据结果进行比较发现,IC-HRMS与LC-MS在很大程度上具有互补性,将二者结合起来可提供一个更全面的寡糖表征体系。IC-MS技术在糖类检测领域的应用总结见表5,其中IC-MS/MS法不仅可以较好地分离各种糖类化合物,还可以获得准确的定量分析结果,是目前食品糖类分析领域中较为理想的方法;IC-HRMS可以提供更为精确的分子质量,因此其在糖类的准确定性方面更为可靠。

**表5:** IC-MS技术在糖类检测中的应用

Analytes	Sample types	IC separation column	Elution solvents and elution program		LODs	LOQs	Method
Erythritol, xylitol, D-sorbitol, D-mannitol, lactitol and maltitol	mirin, vinegar, fruit juice, honey, biscuit and so on	CarboPar MA1 (250 mm× 4 mm)	NaOH solution; isocratic elution	0.28-4.14 mg/kg		0.98-13.56 mg/kg	IC- MS^1[44]^
Galactose, glucose, sucrose and lactose	infant formula milk powder and dairy products	CarboPac PA1 (250 mm× 2 mm)	KOH solution; gradient elution	galactose: 1.76 μg/L; glucose: 3.15 μg/L; sucrose: 23.4 μg/L; lactose: 26.4 μg/L		galactose: 5.85 μg/L; glucose: 10.5 μg/L; sucrose: 78.1 μg/L; lactose: 88.0 μg/L	IC- MS/MS^[45]^
Arabinose, galactose, glucose, sucrose, xylose, mannose, fructose and ribose	coffee beans	CarboPac PA20 (150 mm× 3 mm)	KOH solution; gradient elution	0.01-0.05 mg/L		0.03-0.2 mg/L	IC- MS/MS^[46]^
Glucosylglycerol, sucrose and carbohydrates	cyanobacteria	CarboPac PA20 (150 mm× 0.4 mm)	KOH solution; gradient elution	glucosylglycerol: 0.006 mg/L; sucrose: 0.02 mg/L; carbohy- drates: 0.03 mg/L		glucosylglycerol: 0.02 mg/L; sucrose: 0.07 mg/L; carbohy- drates: 0.1 mg/L	Cap IC- MS/MS^[47]^
Oligosaccharides	chardonnay grape marc	CarboPac PA300 (250 mm× 2 mm)	NaOH solution and sodium acetate; gradient elution	-		-	IC- HRMS^[48]^

### 2.2 药物分析

IC-MS技术在食品安全领域的应用居多,而在药物分析方面的应用则相对较少。目前IC-MS技术主要用于药物杂质分析、药物降解产物鉴定和血药浓度测定。Xiang等^[49]^用IonPac AS5A色谱柱(150 mm×4 mm)进行分离,使用配备了ESI源的三重四极杆质谱仪进行定性,成功鉴定出了新型抗惊厥药物托吡酯片中的有机杂质和无机杂质,包括乳酸盐、乙醇酸盐、氯化物、甲酸盐、硫酸盐和草酸盐。Ahrer等^[50]^采用IC-MS^1^技术,以200 mmol/L甲酸水溶液-乙腈(4∶6, v/v)作为淋洗液,在IonPac CS 14色谱柱(250 mm×4 mm)上进行分离,在一种降胆固醇药物中鉴定出了3种己基三甲基铵的甲氧基和羟基衍生物杂质,LOD为10 μg/mL。Lewis等^[51]^将甲磺酸(2~50 mmol/L)作为淋洗液,在IonPac CS20色谱柱(250 mm×2 mm)上进行梯度洗脱,在ESI^+^模式下对药物合成反应物中甲胺等12种生物胺进行了测定,并采用柱后添加0.05%甲酸和乙醇的方式来提高检测灵敏度和定性准确度,方法LOD为0.0036~0.08 mg/L。Corry等^[52]^使用IC-MS^1^技术,以30 mmol/L KOH为淋洗液,在IonPac AS11-HC色谱柱(250 mm×2 mm)上进行分离,准确、灵敏地测定了原料药2-丁炔酸合成过程中可能存在的乙酸盐、丙酸盐、甲酸盐、丁酸、巴豆酸盐和戊酸等有机酸杂质,所得到的LOQ为1~5 μg/L,相对标准偏差(RSD)为4%~8%。在血药浓度测定方面,Garcia等^[53]^在IonPac AS18色谱柱(150 mm×2 mm)上利用KOH溶液进行洗脱,采用IC-MS/MS法分析了马血浆中6种禁用的双膦酸盐,与使用液相色谱-串联质谱(LC-MS/MS)相比,该方法避免了耗时的衍生化程序;实验结果表明,6种双膦酸盐的LOD为0.2 μg/L,比使用LC-MS/MS方法所获得的LOD(1 μg/L)低了5倍。最近,Wong等^[54]^采用IC-HRMS技术,开展了马血浆中10种双膦酸盐和三焦磷酸肌醇(ITPP)的测定研究,所得到的LOQ低至μg/L级水平,并成功用于马匹中双膦酸盐的残留量监控。综上,IC-MS技术可用于检测和定量分析药物中的杂质、药物合成过程中的副产物和降解产物等,能够显著提高检测的灵敏度和选择性,实现药物的有效分析和杂质鉴定,为药物研发和质量控制提供重要支撑。

### 2.3 代谢组学

人类代谢组由数百种极性代谢产物组成,它们在许多代谢和调节途径中都发挥着关键作用,因此代谢组学研究有助于更好地理解生物体的代谢调控机制,为健康管理和疾病诊断提供重要依据。随着HRMS技术的不断发展和IC-MS技术的进步,HRMS预测小分子结构式的能力越来越强,用于代谢组学的鉴定也越来越广泛^[55,56]^。Wang等^[57]^使用Cap IC-HRMS技术对头颈癌细胞中的阴离子代谢物进行了全面的代谢组学分析,结果表明,与亲水作用色谱-高分辨质谱(HILIC-HRMS)相比,Cap IC-HRMS技术对许多代谢物的检测灵敏度都能够提高约100倍;同时,利用Cap IC-HRMS技术所检测到的目标物覆盖率与使用高效液相色谱-高分辨质谱(HPLC-HRMS)和HILIC-HRMS得到的检测结果之间存在部分重叠,此外利用Cap IC-HRMS技术能够鉴定出更多的代谢物(25种其他代谢物),该工作也引发出了多个将IC-MS技术应用于高极性和离子型代谢物的靶向分析研究^[58⇓⇓⇓⇓⇓⇓⇓-66]^。例如,Hu等^[58]^基于IC-HRMS技术,采用同位素内标法测定了肿瘤细胞中丙酮酸、琥珀酸、苹果酸、柠檬酸、富马酸和*α*-酮戊二酸等6种极性代谢物,LOD低至μmol/L水平;Petucci等^[59]^针对小鼠肌肉中的28种极性有机酸,使用KOH溶液在IonPac AS-11-HC色谱柱(250 mm×2 mm)上进行梯度洗脱分离,采用三重四极杆质谱、ESI^-^模式测定,所得到的28种极性有机酸的LOQ为0.25~50 μmol/L。随着毛细管色谱技术的不断发展,Cap IC-HRMS的应用也越来越广泛。Hirayama等^[67]^开发了一种基于双同轴电喷雾电离源技术的Cap IC-HRMS新方法,以用于阴离子代谢组的分析,该方法可减少因柱后添加淋洗液改性剂而产生的背压影响,明显提高了目标代谢物的峰值分辨率;通过对毛细管色谱柱类型和改性剂进行优化,该方法成功分离出了44种阴离子代谢产物(包括有机酸、糖磷酸酯和核苷酸等),并成功监测到了由肿瘤坏死因子-*α*(TNF-*α*)诱导的癌症细胞培养液中阴离子代谢产物的变化,鉴定出了105种代谢物。Hayasaka等^[68]^通过超速离心法收集小型细胞外囊泡(EVs),利用Cap IC-HRMS和HPLC-HRMS分析小型EVs的亲水性代谢产物,并对不同氧浓度培养下的胰腺癌细胞(PANC-1)以及从细胞中释放出的小型EVs进行了全面的代谢组学分析,结果观察到小型EVs的代谢组学特征在缺氧应激下会发生变化,同时该研究还发现参与血管生成的代谢物种类明显增加。HILIC、毛细管电泳以及气相色谱-高分辨质谱(GC-HRMS)通常是鉴定离子和极性代谢物的主要方法,但这些方法仍然无法覆盖某些离子型或强极性代谢物,而Cap IC-HRMS技术能够为代谢组学研究提供一个强大的分析平台。

### 2.4 临床中毒分析

IC-MS技术在临床中毒分析领域也发挥着重要作用。临床中毒主要涉及烷基膦酸盐、次膦酸盐、草甘膦、百草枯、敌草快、3-硝基丙酸和茚满二酮类鼠药等^[69⇓⇓⇓⇓⇓⇓⇓⇓⇓⇓⇓⇓-82]^。烷基甲基膦酸(AMPAs)和甲基膦酸(MPA)是神经毒剂的水解产物,然而多数AMPAs具有强极性和高度亲水性,在普通液相色谱中不被保留,难以准确分析。因此,Baygildiev等^[71]^使用自制的PS-DVB阴离子交换柱(100 mm×4 mm)进行分离,在对尿液样品进行固相萃取净化后,使用同位素内标-IC-MS/MS法同时测定了人体尿液中的神经毒剂标记物AMPAs和MPA,并取得了满意的效果。Otsuka等^[72]^采用IonPac AS11-HC色谱柱(250 mm×2 mm)进行分离,建立了尿液和血清中6种AMPAs的IC-MS/MS测定方法。Schütze等^[73]^使用IonPac AS24色谱柱(250 mm×2 mm)进行分离、KOH溶液进行梯度洗脱,建立了尿液中草甘膦及6种磷酸二烷基酯代谢物的IC-MS/MS测定方法,所得LOD为0.2~0.8 μg/L。对血液中的草酸盐含量进行测定有助于原发性高草酸尿患者的诊疗,Stokes等^[74]^采用多种方法测定了原发性高草酸尿症患者血液中的草酸盐含量,结果发现,不同检测方法对血液中草酸盐含量的测定结果存在较大差异,其中与GC-MS法相比,IC-MS^1^具有更好的灵敏度(LOQ<27 μg/L);同时对于肾功能恶化的原发性高草酸尿症患者,应由同一家实验室采用同一检测方法进行检测,以避免不同检测结果之间的差异。百草枯和敌草快是常用的除草剂,张秀尧等^[75]^用纯水对血浆和尿液样品进行稀释后,直接使用混合型聚合物和弱阳离子交换填料进行固相萃取净化,并采用阳离子交换色谱进行分离,建立了血浆和尿液中百草枯和敌草快的IC-MS/MS测定方法,实验结果表明,血浆和尿液中百草枯和敌草快的LOD分别为0.3 μg/L和0.2 μg/L。3-硝基丙酸(3-NPA)是一种由曲霉属和青霉属等真菌产生的有毒代谢产物,可以从变质的甘蔗中分离获得。张晓艺等^[76]^使用高氯酸进行超声提取、固相萃取法进行净化,以IonPac AS 19色谱柱(250 mm×2 mm)作为分析柱、KOH溶液作为淋洗液进行梯度分离,在ESI^-^源、MRM模式下,使用基质加标标准曲线进行定量,建立了血浆和尿液中3-NPA的IC-MS/MS检测方法,所得到的LOD为0.1 μg/L。茚满二酮类杀鼠剂在强碱性淋洗液中可以解离成阴离子,因此可以采用IC-MS技术对其进行测定^[77⇓⇓⇓⇓-82]^。异杀鼠酮是一种茚满二酮类杀鼠剂,Cai等^[79]^将含有10%甲醇的KOH溶液作为洗脱液,使用Ionpac AS11色谱柱(250 mm×4 mm)进行分离,建立了血清中异杀鼠酮的IC-MS/MS测定方法,该方法能够满足临床标本中异杀鼠酮的定量测定需求。Chen等^[80]^建立了血清中茚满二酮类杀鼠剂的IC-MS/MS检测方法,该方法使用甲醇提取血清样品,样品经固相萃取净化后在IonPac AS11色谱柱(250 mm×4 mm)上进行分离,在MRM模式下进行测定,实验结果表明4种茚满二酮类杀鼠剂的LOQ为0.2~0.5 μg/L。Jin等^[81]^建立了免试剂离子色谱-电喷雾质谱(RFIC-ESI-MS)同时测定动物肝组织中敌鼠酮、氯鼠酮、杀鼠酮和异杀鼠酮等4种茚满二酮类抗凝血灭鼠剂的方法,该方法利用10%甲醇乙腈将灭鼠剂从动物肝组织匀浆中提取出来,之后利用固相萃取方法进行净化,在IonPac AS11色谱柱(250 mm×4 mm)上进行分离,用含10%甲醇的KOH溶液进行梯度洗脱,结果表明,4种灭鼠剂的LOQ为0.2~1.0 μg/kg。Ouyang等^[82]^使用IonPac AS11色谱柱(250 mm×4 mm)进行分离,利用含10%甲醇的KOH溶液进行洗脱,基于离子色谱-离子阱-电喷雾质谱(IC-IT-ESI-MS)技术建立了血浆中氯鼠酮的测定方法,该方法可用于血浆中氯鼠酮浓度的临床诊断。综上所述,IC-MS技术在强极性、有毒化合物的临床分析中发挥着重要作用,已成为临床中毒分析领域的关键支撑技术。

## 3 结论和展望

食品分析领域存在着大量强极性和可电离物质的检测需求,这为IC-MS技术的应用提供了广阔的空间,IC-MS/MS在其中仍将发挥重要的作用。随着高通量分析需求的不断提升以及多组分分离要求的不断提高,Cap IC-MS/MS和Cap IC-HRMS的应用会得到进一步推广,特别在食品中糖类的识别分析、有机酸的痕量分析以及食品组学分析中将大有作为。在药物分析领域,针对药物生产过程中所带入的强极性或可电离杂质、反应生成的副产物、药物贮存运输过程可能产生的降解产物、药物在体内的代谢产物等进行研究具有重要意义。随着新药研制生产和临床药物不良反应研究的不断深入,IC-MS/MS、Cap IC-MS/MS和Cap IC-HRMS将发挥越来越重要的作用,相关的应用研究将是药物分析领域的一个重要研究方向。在代谢组学领域,由于多数代谢产物具有强极性,在传统的液相色谱中难以得到较好的保留和分离,离子色谱特别是毛细管离子色谱柱的出现,为代谢组学的研究提供了更多的可能性。随着肿瘤细胞代谢物研究的逐渐深入,可识别的强极性代谢物种类将越来越多,Cap IC-HRMS可能会在肿瘤细胞的调控机制研究中发挥关键作用。在临床中毒检测方面,针对生物样本中的毒物原形及其强极性代谢产物的检测,IC-MS/MS、Cap IC-MS/MS和Cap IC-HRMS技术将发挥重要的作用。

随着仪器制造技术的不断发展,IC-MS技术的灵敏度和分辨率得到了显著提高,特别是新型毛细管离子色谱柱的性能得到了不断提升,为离子色谱与各类质谱的联用创造了极佳的接口条件。未来,Cap IC-HRMS将是IC-MS技术的一个重要发展方向。目前,IC-MS技术的瓶颈是淋洗液的除盐,尽管与窄径离子色谱柱、毛细管离子色谱柱及抑制器进行联用能够较好地解决无机盐在质谱离子源中的沉积及毛细管堵塞问题,但仍会有极少量无机盐进入质谱离子源,未来挥发性淋洗液的开发可能会成为IC-MS技术的一个重要研究方向。同时,现代分析面临着代谢组学等大量数据的处理需求,IC-MS技术同样需要开发出更高效和自动化的数据处理方法及分析方法,以提高数据的利用率,这也是未来的研究工作重点。

综上所述,未来IC-MS技术将会在生命健康领域取得新的突破,为生命健康领域的分析和研究提供更强大的支持和推动力。
